# Spine Buddy® Supportive Pad Impact on Single-Leg Static Balance and a Jogging Gait of Individuals Wearing a Military Backpack

**DOI:** 10.2478/hukin-2014-0110

**Published:** 2014-12-30

**Authors:** John Ward, Jesse Coats, Amir Pourmoghaddam

**Affiliations:** 1 Department of Physiology and Chemistry, Texas Chiropractic College.; 2 Department of Clinical Specialties, Texas Chiropractic College.; 3 Memorial Bone & Joint Clinic.

**Keywords:** gait, biomechanics, locomotion, load carriage, balance

## Abstract

The Spine Buddy® supportive pad was developed to be inserted underneath military backpacks to help disperse the heavy load of the backpack. The purpose of this study was to determine the impact the additional supportive pad had on static balance and a running gait while wearing a military backpack. Forty healthy subjects (age= 27.5 + 5.6 yrs, body height= 1.78 + 0.06 m, body mass= 86.5 + 14.0 kg: mean + SD) participated in a static single-leg balance test on a force plate with each lower limb while wearing a 15.9 kg military backpack for 30 s. Following this, participants were randomized to one of two interventions: 1) Intervention, which wore the Spine Buddy® supportive pad underneath their backpack or 2) Control, with no additional supportive pad. Post-intervention measurements of static single-leg balance were then recorded. Afterwards, a similar pre vs post testing schedule and randomization scheme was used to test the impact of the supportive pad on a 5 mph jogging gait using Vicon® cameras. Within-group data were analyzed with a 2-way repeated measures ANOVA. Statistically significant differences were not seen between the control and experimental group for balance and gait variables. Preliminarily, this suggests that the Spine Buddy® supportive pad causes no deleterious effect on static balance and a jogging gait in 18–45 year-old asymptomatic individuals.

## Introduction

Low back pain (LBP) is common. Research has shown that LBP is the fifth most common reason for all physician visits (Hart et al., 1995; Jarvik and Deyo, 2002) and it is the second leading cause of disability in persons under 45 years of age (CDC, 2001). Surveys have found that 7.6% of people annually reported at least one occurrence of an episode of severe acute LBP (Carey et al., 1996). LBP is economically expensive for multiple reasons. In 1998 the cost of treating LBP was $26.3 billion dollars in the US (Chou et al., 2007) and those costs have continued to rise both locally and globally (Dagenais et al., 2008; Crow and Willis, 2009; Hoy et al., 2010; Lambeek et al., 2011). In addition to these medical economic costs there are also societal economic costs due to lost work productivity from absenteeism (Murtezani et al., 2010; Hoogendoom et al., 2002; Steenstra et al., 2009; Infante-Rivard and Lortie 1997) and presenteeism (Aronsson et al., 2000), as well as the more subjective costs of reduced quality of life for the duration of LBP (Dagenais et al., 2008; Bernstein et al., 2004). Absenteeism is when a person does not go to work because they are injured. Studies have demonstrated that annually 2% of individuals miss work due to low back pain (Punnett et al., 2007). Presenteeism is when a person who is injured shows up to work, but he/she is not able to perform their job effectively because of his/her injury (Bernstein et al., 2004), and this results in reduced work productivity (Johns, 2010; Goetzel et al., 2004).

Musculoskeletal injuries are common in the military (Glad et al., 2012; Knapik et al., 2007; Kaufman et al., 2000; Knapik et al., 2004; Daube 1969; Bessen et al., 1987; Mäkelä et al., 2006). The primary reason for medical evacuation of US military personnel in Iraq and Afghanistan was musculoskeletal injuries (Cohen et al., 2010), and musculoskeletal injuries remain a common reason for active duty military to utilize healthcare services (Waitzkin and Noble, 2009; Mattila et al., 2006; Jones and Knapik, 1999). Musculoskeletal injuries also represent a common reason that leads to premature discharge from military service (Taanila et al., 2010). Military soldiers, particularly warfighters, routinely wear heavy backpacks (Knapik et al., 1996; Knapik et al., 1997) and are at risk of developing low back pain due to the strenuous physical nature of their work (Taanila et al., 2010; Taanila et al., 2009; Jennings et al., 2008). Research even suggests that military service is a predictor of low back pain later in life (Mattila et al., 2009; Hellsing and Bryngelsson, 2000).

Carrying heavy loads requires greater energetic costs that are associated with the increased force necessary to move the body (Marsh et al., 2006; Ellerby and Marsh, 2006; Bastien et al., 2005; Grenier et al., 2012; Griffin et al., 2003; Pandolf et al., 1977). When carrying a military backpack for long road marches it has been shown to induce neurologic impairment (Clarke et al., 1955; Blacker et al., 2010; Grenier et al., 2012). Additionally, it can result in deficiences in dynamic balance and posture (Oh and Choi, 2007; Matsuo et al., 2008; Costello et al., 2012), which can render individuals at risk of spinal disorders (Whittfield et al., 2001) as well as falls or other injuries (Knapik et al., 1996; Simpson et al., 2011; Gefen, 2002; Murdoch and Hubley-Kozey, 2012; Parijat and Lockhart, 2008). Any action to reduce the prevalence and incidence of back pain in soldiers should be pursued.

Various countermeasures can be used to reduce prevalence of back pain in the military, to include: strengthening exercises (Balague et al., 1999; Burton et al., 1996) and more ergonomically effective equipment. One such proposed ergonomic device is the Spine Buddy® supportive pad. It is a pad that can be added onto a military backpack between the pack and the wearer. The supportive pad was developed to help distribute the weight of heavy military backpacks more evenly on the body frame instead of primarily on the shoulders.

As with any new device to be considered for use by the military it must undergo extensive testing. The purpose of this study was to determine if the Spine Buddy® supportive pad negatively impacted static balance or jogging gait parameters in asymptomatic individuals.

## Material and Methods

This study was reviewed and approved by the Texas Chiropractic Institutional Review Board for human subjects in accordance with the Declaration of Helsinki. All subjects were provided a written and oral explanation of the study procedures prior to participation. This trial was registered with the University hospital Medical Information Network Clinical Trials Registry (UMIN-CTR), trial number: UMIN000014666.

### Study Design and Setting

This was a single-blind, randomized, controlled study of the immediate impact that the Spine Buddy® supportive pad had on static balance and jogging kinematics in asymptomatic individuals wearing a 15.9 kg military backpack. Specific aims were to determine if using the Spine Buddy® supportive pad resulted in diminished static balance or any negative changes in jogging kinematics.

**Figure 1:**
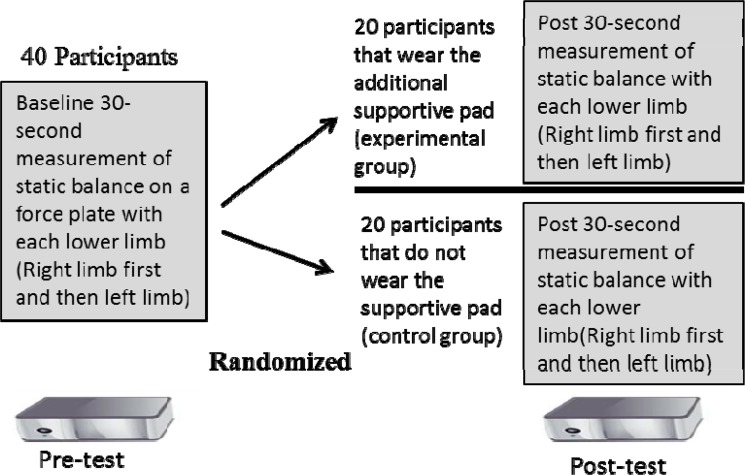
Experimental design for the force plate single-leg balance test. This test was performed with each lower limb, pre vs post. As soon as participants finished their entire balance test schedule they engaged in a similarly structured protocol schedule for their pre vs post jogging kinematic analyses, except they ran at 5 mph for 90 s during each test session (pre vs post). Once participants were assigned to a given study group (experimental or control) they stayed in that group for the whole study.

**Figure 2:**
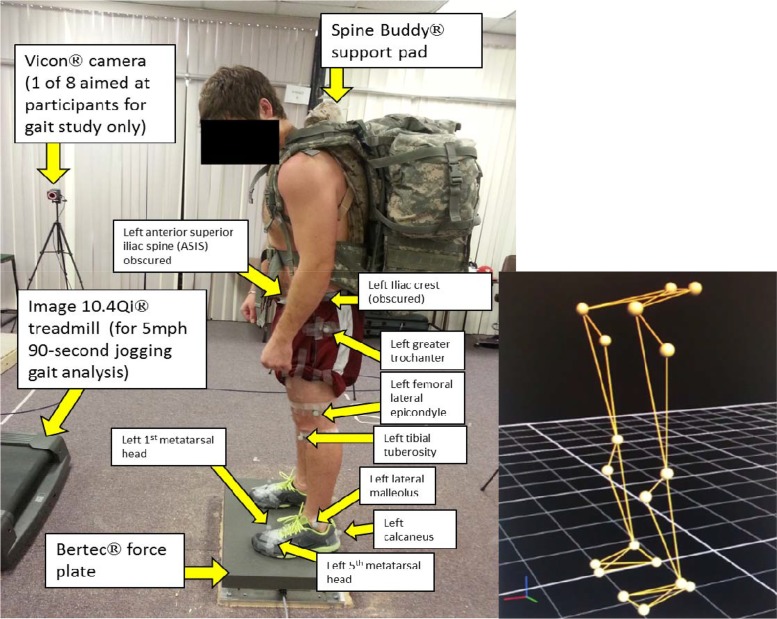
*Illustration of a study participant and a sample computer model based on silver reflective marker data extraction using the Vicon® imaging system. Only the left side of the participant’s silver reflective markers are labelled in this diagram to avoid image clutter*.

As shown in Figure 1, forty participants initially engaged in a 30 s analysis of their static single-leg balance on each lower limb while wearing a military backpack. Next participants were randomized to one of two interventions: 1) Intervention, which wore the Spine Buddy® supportive pad underneath their backpack or 2) Control, with no additional supportive pad. Afterwards, the study participants engaged in a post-intervention analysis of static single-leg balance on each leg. Following this, the subjects engaged in a baseline 90 s jogging kinematic analysis at 5 mph without a supportive pad. This was done utilizing a Vicon® motion analysis camera system (Vicon, Centennial, CO, USA) as shown in Figure 2. Then half of the participants were randomized to wear the Spine Buddy® supportive pad underneath their backpack. Next, all study participants repeated their 90 s 5 mph jogging gait analysis post-intervention. The subjects in the control group engaged in testing twice without the additional support pad to demonstrate test-retest data range variability.

### Participants

Asymptomatic college students were recruited for this study. All study applicants provided informed written consent on college-approved documents. They were then screened against inclusion and exclusion criteria. Forty apparently healthy individuals (range= 22–49 years-of-age) that met the inclusion/exclusion criteria participated in this twenty-minute one-visit study (Table 1). No participants were excluded from this study due to violating the exclusionary criteria. These criteria were discussed with students in multiple classes and likely discouraged individuals that would not qualify from attempting to contact researchers.

### Inclusion/exclusion criteria

Inclusion criteria were: 1) between the ages of 18–50 years old, 2) answering “no” to all exercise contraindication sections on a Physical Activity Readiness-Questionnaire (PAR-Q), 3) they did not engage in strenuous exercise the day of the study, and 4) they provided their informed written consent. Study participants with any of the following were excluded from the study: 1) diagnosis of any lumbar, sacral, hip, or lower limb pathology that would prevent them from jogging, 2) severe neurological conditions which would impact their gait (e.g., type II diabetes, Parkinson’s disease, Traumatic Brain Injury, Dementia, Stroke, Epilepsy, Multiple Sclerosis, Myasthenia Gravis, Huntington’s disease, etc.), 3) history of alcohol abuse, 4) any health condition that would impair their ability to jog up to 5 mph, 5) visual impairment that would render jogging on a treadmill dangerous for them, 6) hypertonia, 7) use of a cane or similar assistive device, 8) taking medications that alter motor function (e.g., acetylcholine-esterase inhibitors, L-dopa agonists, dopa-antagonists, or neuroleptics), 9) botulinum injection in their lower limb muscles within the past six months, 10) presence of severe pain in their lower limbs that they would rate greater than a 3 on a 0 to 10 Numeric Rating Scale (NRS), 11) vertigo or history of falls within the past 60 days, or 12) any prior bone or muscle-related surgeries.

### Randomization and blinding

A computer-generated randomized intervention list was created before the study began. That list determined if a participant would be assigned to either group (experimental vs control). The biomechanics researcher who analyzed the force plate and motion capture data were blinded as to group designation. He was only told that he would be provided with balance and kinematic data from two distinct study groups and that he needed to determine if any unique differences existed between any of the groups’ pre versus post data.

### Intervention

**Figure 3:**
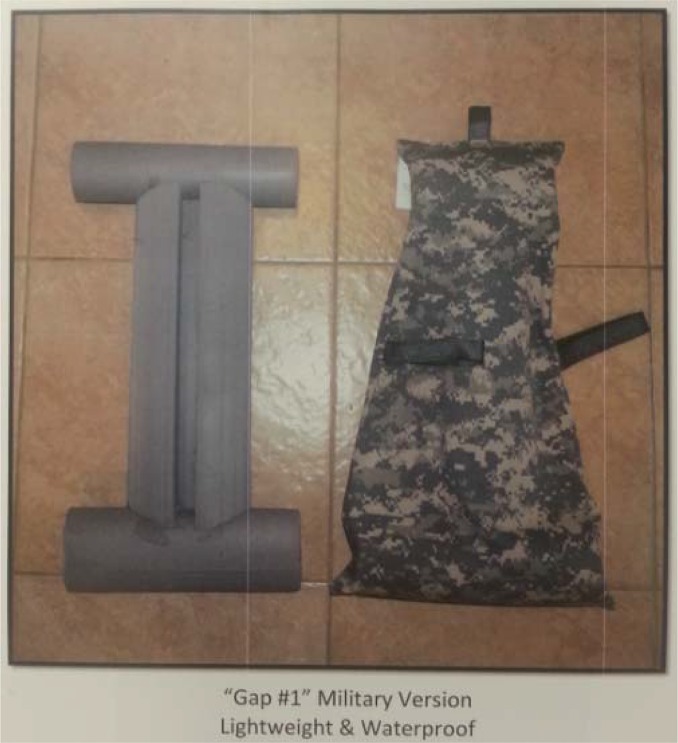
Illustration of the Gap 1 waterproof Spine Buddy® pad (a) left picture, 1340 polyurethane foam core insert, (b) right picture, the camouflage waterproof nylon insert cover with straps to secure to a backpack frame.

The intervention phase of the study involved placing the Spine Buddy® supportive pad (Figures 2–3) underneath a military backpack for half of the participants and adjusting the shoulder straps accordingly for participant comfort. The added supportive pad was designed to dampen force exerted on the spine while wearing a heavy military backpack. The impact adding this type of supportive pad, due to its small size, to a typical military backpack had not been studied before. Researchers hypothesized that the supportive pad would not hinder static balance or a jogging gait while wearing a military backpack. Study participants were not blinded to the study intervention. The Spine Buddy® supportive pad utilized in this study was the camouflage Gap 1 waterproof model (International Neck & Back Cushion Enterprises, Humble, TX, USA) with a gap down the middle of the pad, anteriorly, to ergonomically accommodate the spinous processes of the thoracic spine. The support pad was composed of a woven Nylon cover with an internal 1340 polyurethane foam core. The foam had the following attributes as determined by the ASTM D-3574 test method: 1.30 ± 0.1 lbs/cu ft density, 12 min lbs/inch tensile strength, 1.5 min lbs/sq inch tear strength, and 160% elongation. The foam was made by Microcell (Hyannis, MA, USA).

### Single Leg Stance Test

Participants stood on top of a Bertec 4060-NC force plate (Bertec Corp., Columbus, OH, USA) as illustrated in Figures 1–2. The force plate data were recorded directly through Vicon®. Participants were instructed that they would be standing on their right foot as long as they could without falling for up to 30 s. After this they would repeat the same test by standing on their left foot as long as they could without falling for up to 30 s. Furthermore, half of the participants were randomized to wear the Spine Buddy® supportive pad underneath their backpack. Then all participants engaged in the same single-leg post-testing analysis of static balance for their right and left lower limbs on the force plate. This was performed to discern if the added pad impacted static single-leg balance. Force plate data were exported from the Vicon® system and analyzed with Matlab® to quantify how long the participant could stand on either limb before losing their balance and falling, if at all, under the pre and post-testing conditions. For data analysis purposes, participant data from the right and left lower limb stance time were averaged together per test session, pre vs post. Researchers chose to test both lower limbs with the single-leg balance test to statistically rule out leg dominance as a study covariate.

### Baseline Preparation and Kinematic Recording

Trained research assistants placed 18 silver 19 mm MoCap solutions (MoCap solutions, Huntington Beach, CA, USA) reflective markers on the participant’s lower body using surgical tape. Reflective markers were placed on the following anatomic landmarks during this study bilaterally: anterior superior iliac spine (ASIS), iliac crest, greater trochanter of the femur, lateral epicondyle of the femur, tibial tuberosity, lateral malleolus, posterior calcaneus, top of the fifth metatarsal head, and top of the first metatarsal head (Figure 2), with a marker set and model as described by Robertson et al. (2004).

Prior to the participant arriving at the lab each day the Vicon® system was calibrated as suggested by the manufacturer. Once the participant was dressed properly in non-reflective clothing and all of the reflective silver markers were in place they stood on top of the Image 10.4Qi® treadmill (Sears, Hoffman, IL, USA) for their baseline 10 s computer calibration model generation. Next the participant was instructed that they would be jogging as they normally would at a velocity of 5 mph. A research assistant started the treadmill at the same time as another researcher began recording data with the Vicon® system. The lab’s Vicon® MX system consisted of 8 infrared Bonita 0.3 megapixel cameras. Kinematic data were recorded at 100 Hz. The displacement of the 18 silver reflective markers over time was recorded. At the conclusion of 100 s the researcher operating the Vicon® computer system stopped the recording and then the treadmill was stopped. The study participant was not given any indication of when the treadmill would be stopped prior to the examiner finishing his computer data recording. Immediately after the 100 s recording was made the initial 10 s of data were clipped from the total data to remove any initial steps as the participant became acclimated to the treadmill velocity upon beginning the test. Following the baseline 90 s of data collection the participant then carefully stepped off of the treadmill. Afterwards, half of the participants were randomized to wear the Spine Buddy® supportive pad underneath their backpack. Then all participants engaged in the same gait test as a post-test. After participants completed the study protocol, those who wore the Spine Buddy® supportive pad were asked if they preferred wearing the extra pad or not during the gait analysis and responses were tallied.

### Kinematic Post-data Processing

The data were processed using a customized Matlab script (Mathworks, USA R2007a). Force plate data were used to determine how long the participant kept their balance before falling, if at all. The kinematic data were analyzed to calculate characteristics of movement for each participant. In the current study researchers investigated the changes in the functional active range of motion of the hip angle, knee angle, and ankle angle as a result of the intervention. In addition, stance time, percent stance time (duration one foot was on the ground in relation to the gait cycle), step length, and stride length bilaterally were calculated. Right and left limb individual data were then merged for statistical analysis.

Approximate Entropy (ApnEn), a measure of gait variability, was additionally determined for each joint (Myers et al., 2010; Buzzi et al., 2003; Pincus, 1999). In healthy individuals there is a certain amount of acceptable variability that represents a normal (healthy) gait pattern. However, highly variable gait patterns are typically indicative of some type of pathology or loss of coordination (Myers et al., 2010), which may render a person at risk of falling (Maki, 1997). Values near “0” represent a stable gait, while values near “2” represent a very unstable gait.

### Statistical Analysis

To analyze the kinematic and balance data researchers utilized a two-way repeated-measures ANOVA considering a test session (pre-test, post-test) and group (support pad, no pad) as subject factors. The Mauchly’s test was applied to check the sphericity assumption of the repeated-measures ANOVA and the Greenhouse-Geisser correction was utilized during instances of sphericity violation. A Bonferroni post-hoc test was conducted on statistically significant data amongst all ANOVAs to determine which condition was significant. The alpha level of p < 0.05 was considered statistically significant for all tests. Study data are illustrated in Tables 1–3. The data were analyzed using SPSS 20.0 (SPSS Inc., Chicago, IL, USA).

## Results

Table 2 illustrates the grouped attributes of participants from the static single-leg balance test. No statistically significant within-group changes occurred in either group. Both groups had almost perfect scores under each condition.

Table 3 illustrates kinematic data from the two study groups. There was no statistically significant within-group difference in either group for kinematic parameters analyzed for the pre vs post 5 mph jogging gait analysis.

Lastly, 70% of participants who wore the Spine Buddy® supportive pad reported preferring the pad to not wearing the extra pad during the jogging kinematic analyses.

## Discussion

The changes in jogging kinematics in response to wearing the Spine Buddy® supportive pad were small and did not reach a statistically significant level. This study’s original hypothesis was that participants’ single-leg static balance and their jogging gait would be minimally impacted by an extra supportive pad. Additionally, researchers believed that participants would prefer an extra pad as opposed to not wearing an extra pad because of the physical support the pad may have provided the spine. Understanding how the pad would impact their jogging gait is critical to determine if the pad would negatively affect runner economy. For example, if the weight was held too high on the back due to the supportive pad that may result in more side-to-side motion as soldiers run. Measuring gait kinematics can determine if wearing an additional pad negatively impacts runner performance.

The implications of the study’s findings suggest that wearing an additional small supportive pad does not have a negative impact on static balance and a jogging gait. As a result, the choice of a soldier to wear an additional pad would be more of an issue of comfort. Studies have shown that carrying loads more posteriorly negatively impacts the center of gravity (Data and Ramanathan, 1971; Legg, 1985; Woodhull et al., 1985). Ultimately, the addition of a small supportive pad did not appear to negatively impact balance amongst participants. The extent to which the device can reduce the prevalence and incidence of back pain amongst warfighters should be studied further.

Military soldiers often develop musculoskeletal health conditions (Glad et al., 2012; Knapik et al., 2007) and depending on their severity they can lead to premature discharge (Taanila et al., 2010). Several studies have demonstrated that soldiers often develop low back pain (Taanila et al., 2010; Taanila et al., 2009; Jennings et al., 2008), particularly as they age (Mattila et al., 2009; Hellsing and Bryngelsson, 2000). Any equipment that can help reduce the prevalence and incidence of low back pain for the military should be studied.

Future directions of research should analyze how firing of weapons in the prone position will be impacted by soldiers wearing a military backpack with the Spine Buddy® supportive pad attached. Also due to the way the supportive pad extends to the base of the posterior neck, studies should be performed on how the pad impacts whiplash in military vehicles. Lastly, research should be performed comparing different compressive core densities of the supportive pad to see how they impact performance of common military tasks under various weather conditions (e.g., rain, hot weather, snow, etc.).

## Limitations

Researchers did not recruit a set number of participants based on a power analysis due to this intentionally being a pilot study. Following a post-hoc power analysis using G*Power version 3.1.3 (Universität Kiel, Germany) (Faul et al., 2009; Erdfelder et al., 1996) researchers determined study power was 0.5645. This analysis was in accordance with a desired moderate effect size of 0.5, α of 0.05, and 20 participants per group condition compared. In consideration of this analysis, the current study was underpowered and the possibility of Type II error exists. Ideally, in order to have a power of 0.80, researchers would need 34 participants per group. Despite this, it is normal in exercise science research to engage in underpowered studies involving 10–20 participants per compared study group to observe data trends (Crecelius et al., 2011; Froyd et al., 2013; Gavin et al., 2007; Kim et al., 2014; Paschalis et al., 2013)

Participants were running on a treadmill at a steady velocity and running performance would likely be different than what would occur on uneven ground outdoors as discussed by Kluitenberg et al. (2012). The impact an additional supportive pad would have on cushioning the load carried by soldiers as they traversed cross-country and any impact it would have on their center of gravity warrants further review.

This study only informs us as to the immediate impact wearing the Spine Buddy® supportive pad had on specific gait parameters in asymptomatic individuals. The impact the Spine Buddy® pad would have on injured soldiers with various lower limb musculoskeletal conditions (e.g., shin splints, plantar fasciitis, gunshot wounds) during a similar study remains unclear.

Additionally, this study’s participants were males. The impact this extra supportive pad would have had on females with their slightly different center-of-gravity warrants further review. This study focused on males due to their higher probability of being in a combat situation and wearing heavy military backpacks.

Lastly, the subjects participating in this study were typical college students. Research has shown that a significant number of people in the general public are not physically fit enough to be in the military (Gubata et al., 2011; Niebuhr et al., 2008; Niebuhr et al., 2009; Bedno et al., 2010; Cowan et al., 2011). As a result, the ability to apply these results strictly to military soldiers may be limited.

## Conclusions

There is minimal research into how additional supportive pads worn underneath a military backpack impact static balance and a jogging gait. The focus of this experiment was to determine if wearing an additional supportive pad would negatively impact static balance and a gait, and to measure if participants had a preference for or against the use of an extra support pad. The findings of this study suggest that wearing the Spine Buddy® support pad has no negative impact on single-leg static balance or jogging kinematics during treadmill running. Additionally, the findings of this research demonstrate that participants prefer to use the supportive pad as opposed to not using the pad when jogging with a military backpack.

## Figures and Tables

**Table 1:** Baseline study participants characteristics

	Spine Buddy® group	no extra pad group
	(Experimental group)	(Control group)
Participants	20	20
Age (y)	28.7 + 6.9	26.4 + 4.4
Body Mass (kg)	85.0 + 12.1	88.1 + 15.9
Body Height (m)	1.78 + 0.07	1.79 + 0.06
Body Mass Index (kg/m2)	26.9 + 3.2	27.3 + 3.8

Data listed as mean + SD.

**Table 2:** Results from the 30 s static single-leg balance test comparing the use of the Spine Buddy® supportive pad (experimental group) to no pad (control)

	Spine Buddy® group	no extra pad group
	(Experimental group)	(Control group)
Average single-leg stance time for both lower limbs (s) PRE	29.8 + 0.6	29.7 + 0.7
Average single-leg stance time for both lower limbs (s) POST	29.7 + 0.7	28.6 + 0.8
p	0.941	0.793

Data listed as mean + SD.

**Table 3:** Gait attribute data for the Spine Buddy® supportive pad group and control group jogging at 5 mph for 90 s. HpROM = hip functional range of motion in degrees; KnROM = knee functional range of motion in degrees; AnROM= ankle functional range of motion in degrees; STSec= stance time in seconds; %ST= stance percentage of gait cycle; StepLen= step length in millimeters (25.4 mm= 1 inch); StriLen= stride length in millimeters; HpApnEn= hip approximate entropy; KnApnEn= knee approximate entropy; AnApnEn= ankle approximate entropy. LCI = lower 95% confidence interval in relation to mean difference; UCI = upper 95% confidence interval in relation to mean difference. Spine Buddy® supportive pad group (experimental group)

	Pre		Post					

	Mean	SD	Mean	SD	Mean Diff	LCI	UCI	p
	
HpROM	61.1	6.9	60.4	4.6	0.7	−1.10	2.56	0.396
KnROM	81.0	8.6	80.6	8.9	0.4	−1.34	2.12	0.624
AnROM	55.1	9.8	55.7	9.3	−0.6	−2.73	1.52	0.541
STSec	0.43	0.03	0.43	0.04	0.0	−0.0086	0.0072	0.853
%ST	57.1	1.5	57.3	1.5	−0.02	−0.38	0.07	0.160
StepLen	660.0	68.4	662.8	59.9	−2.8	−23.78	18.24	0.775
StriLen	1406.0	150.0	1408.4	144.6	−2.4	−43.88	38.95	0.897
HpApnEn	0.18	0.27	0.27	0.06	−0.09	−0.03	0.01	0.398
KnApnEn	0.32	0.35	0.34	0.06	−0.02	−0.02	0.02	0.778
AnApnEn	0.38	0.03	0.38	0.04	0.0	−0.31	0.02	0.681

Control group (no additional pad)								

	Pre		Post					

	Mean	SD	Mean	SD	Mean Diff	LCI	UCI	p
	
HpROM	57.7	7.0	57.7	6.1	0.0	−1.89	1.87	0.991
KnROM	81.1	10.9	82.2	9.2	−1.1	−4.32	2.07	0.450
AnROM	51.8	6.1	51.5	5.8	0.3	−1.08	1.72	0.624
STSec	0.43	0.03	0.43	0.03	0.0	−0.0065	0.0107	0.604
%ST	56.6	1.1	56.9	−0.3	−0.3	−0.60	0.00	0.051
StepLen	660.3	66.2	662.1	55.7	−1.8	−13.44	9.72	0.728
StriLen	1361.9	143.1	1366.9	118.0	−5.0	−28.51	18.36	0.640
HpApnEn	0.27	0.05	0.27	0.05	0.0	−0.2	0.03	0.700
KnApnEn	0.33	0.05	0.33	0.04	0.0	−0.2	0.02	0.450
AnApnEn	0.37	0.04	0.34	0.04	0.03	−0.00	0.06	0.624

## References

[b1-jhk-44-53] Aronsson G, Gustafsson K, Dallner M (2000). Sick but yet at work. An empirical study of sickness presenteeism. J Epidemiol Commun H.

[b2-jhk-44-53] Balague F, Troussier B, Salminen J (1999). Non-specific low back pain in children and adolescents: risk factors. Eur Spine J.

[b3-jhk-44-53] Bastien G, Willems P, Schepens B, Heglund N (2005). Effect of load and speed on the energetic cost of human walking. Eur J Appl Physiol.

[b4-jhk-44-53] Bedno S, Lang C, Daniell W, Wiesen A, Datu B, Niebuhr D (2010). Association of weight at enlistment with enrollment in the Army Weight Control Program and subsequent attrition in the Assessment of Recruit Motivation and Strength Study. Mil Med.

[b5-jhk-44-53] Blacker S, Fallowfield J, Bilzon J, Willems M (2010). Neuromuscular function following prolonged load carriage on level and downhill gradients. Aviat Space Environ Med.

[b6-jhk-44-53] Bernstein E, Carey T, Garrett J (2004). The use of muscle relaxant medications in acute low back pain. Spine (Phila Pa 1976).

[b7-jhk-44-53] Bessen R, Belcher V, Franklin R (1987). Rucksack paralysis with and without rucksack frames. Mil Med.

[b8-jhk-44-53] Burton A, Clarke R, McClune T, Tillotson K (1996). The natural history of low back pain in adolescents. Spine.

[b9-jhk-44-53] Buzzi U, Stergiou N, Kurz M, Hageman P, Heidel J (2003). Nonlinear dynamics indicates aging affects variability during gait. Clin Biomech.

[b10-jhk-44-53] Carey T, Evans A, Hadler N, Lieberman G, Kalsbeek W, Jackman A, Fryer J, McNutt R (1996). Acute severe low back pain. A population-based study of prevalence and care-seeking. Spine (Phila Pa 1976).

[b11-jhk-44-53] CDC (2001). MMWR.

[b12-jhk-44-53] Chou R, Qaseem A, Snow V, Casey D, Cross T, Shekelle P, Owens D (2007). Diagnosis and treatment of low back pain: a joint clinical practice guideline from the American college of physicians and the American pain society. Ann Intern Med.

[b13-jhk-44-53] Clarke H, Shay C, Mathews D (1955). Strength decrements from carrying various army packs on military marches. Res Q.

[b14-jhk-44-53] Cohen S, Brown C, Kurihara C, Plunkett A, Nguyen C, Strassels S (2010). Diagnoses and factors associated with medical evacuation and return to duty for service members participating in Operation Iraqi Freedom or Operation Enduring Freedom. A prospective cohort study. Lancet.

[b15-jhk-44-53] Costello K, Matrangola S, Madigan M (2012). Independent effects of adding weight and inertia on balance during quiet standing. BioMed Eng Online.

[b16-jhk-44-53] Cowan D, Bedno S, Urban N, Yi B, Niebuhr D (2011). Musculoskeletal injuries among overweight army trainees: incidence and health care utilization. Occup Med.

[b17-jhk-44-53] Crecelius A, Kirby B, Voyles W, Dinenno F (2011). Augmented skeletal muscle hyperaemia during hypoxic exercise in humans is blunted by combined inhibition of nitric oxide and vasodilating prostaglandins. J Physiol.

[b18-jhk-44-53] Crow W, Willis D (2009). Estimating the cost for patients with acute low back pain: a retrospective review of patient records. J Am Osteopath Assoc.

[b19-jhk-44-53] Dagenais S, Caro J, Haldeman S (2008). A systematic review of low back pain cost of illness studies in the United States and internationally. Spine J.

[b20-jhk-44-53] Data S, Ramanathan N (1971). Ergonomic comparison of seven modes of carrying loads on the horizontal plane. Ergonomics.

[b21-jhk-44-53] Daube J (1969). Rucksack paralysis. J Amer Med Assoc.

[b22-jhk-44-53] Ellerby D, Marsh R (2006). The energetic costs of trunk and distal-limb loading during walking and running in guinea fowl *Numida meleagris*. Muscle energy use as indicated by blood flow. J Exp Biol.

[b23-jhk-44-53] Erdfelder E, Faul F, Buchner A (1996). GPOWER: A general power analysis program. Behav Res Meth.

[b24-jhk-44-53] Faul F, Erdfelder E, Buchner A, Lang A (2009). Statistical power analyses using G*Power 3.1: Tests for correlation and regression analyses. Behav Res Meth.

[b25-jhk-44-53] Froyd C, Millet G, Noakes T (2013). The development of peripheral fatigue and short-term recovery during self-paced high-intensity exercise. J Physiol.

[b26-jhk-44-53] Gavin T, Ruster R, Carrithers J, Zwetsloot K, Kraus R, Evans C, Knapp D, Drew J, McCartney J, Garry J, Hickner R (2007). No difference in the skeletal muscle angiogenic response to aerobic exercise training between young and aged. J Physiol.

[b27-jhk-44-53] Gefen A (2002). Biomechanical analysis of fatigue-related foot injury mechanisms in athletes and recruits during intensive marching. Med Biol Eng Comput.

[b28-jhk-44-53] Glad D, Skillgate E, Holm L (2012). The occurrence and severity of musculoskeletal disorders in Swedish military personnel during peacekeeping operations in Afghanistan. Eur Spine J.

[b29-jhk-44-53] Goetzel R, Long S, Ozminkowski R, Hawkins K, Wang S, Lynch W (2004). Health, absence, disability, and presenteeism cost estimates of certain physical and mental health conditions affecting U.S. employers. J Occup Environ Med.

[b30-jhk-44-53] Grenier J, Millet G, Peyrot N, Samozino P, Oullion R, Messonnier L, Morin J (2012). Effects of extreme-duration heavy load carriage on neuromuscular function and locomotion: a military-based study. PloS ONE.

[b31-jhk-44-53] Grenier J, Peyrot N, Castells J, Oullion R, Messonnier L, Morin J (2012). Energy cost and mechanical work of walking during load carriage in soldiers. Med Sci Sports Exerc.

[b32-jhk-44-53] Griffin T, Roberts T, Kram R (2003). Metabolic cost of generating muscular force in human walking: insights from load-carrying and speed experiments. J Appl Physiol.

[b33-jhk-44-53] Gubata M, Cowan D, Bedno S, Urban N, Niebuhr D (2011). Self-reported physical activity and preaccession fitness testing in U.S. Army applicants. Mil Med.

[b34-jhk-44-53] Hart L, Deyo R, Cherkin D (1995). Physician office visits for low back pain. Frequency, clinical evaluation, and treatment patterns from a U.S. national survey. Spine (Phila Pa 1976).

[b35-jhk-44-53] Hellsing A, Bryngelsson I (2000). Predictors of musculoskeletal pain in men: A twenty-year follow-up from examination at enlistment. Spine.

[b36-jhk-44-53] Hoogendoorn W, Bongers P, de Vet H, Ariens G, van Mechelen W, Bouter L (2002). High physical work load and low job satisfaction increase the risk of sickness absence due to low back pain: results of a prospective cohort study. Occup Environ Med.

[b37-jhk-44-53] Hoy D, Brooks P, Blyth F, Buchbinder R (2010). The epidemiology of low back pain. Best Pract Res Clin Rheumatol.

[b38-jhk-44-53] Infante-Rivard C, Lortie M (1997). Relapse and short sickness absence for back pain in the six months after return to work. Occup Environ Med.

[b39-jhk-44-53] Jarvik J, Deyo R (2002). Diagnostic evaluation of low back pain with emphasis on imaging. Ann Intern Med.

[b40-jhk-44-53] Jennings B, Yoder L, Heiner S, Loan L, Bingham M (2008). Soldiers with musculoskeletal injuries. J Nurs Scholarsh.

[b41-jhk-44-53] Johns G (2010). Presenteeism in the workplace: a review and research agenda. J Organ Behav.

[b42-jhk-44-53] Jones B, Knapik J (1999). Physical training and exercise-related injuries. Surveillance, research and injury prevention in military populations. Sports Med.

[b43-jhk-44-53] Kaufman K, Brodine S, Shaffer R (2000). Military training-related injuries: surveillance, research, and prevention. Am J Prev Med.

[b44-jhk-44-53] Kim E, Gregg L, Kim D, Sherk V, Bemben M, Bemben D (2014). Hormone responses to an acute bout of low intensity blood flow restricted resistance exercise in college-aged females. J Sports Sci Med.

[b45-jhk-44-53] Kluitenberg B, Bredeweg S, Zijlstra S, Zijlstra W, Buist I (2012). Comparison of vertical ground reaction forces during overground and treadmill running. A validation study. BMC Musculoskelet Disord.

[b46-jhk-44-53] Knapik J, Harman E, Reynolds K (1996). Load carriage using packs: a review of physiological, biomechanical and medical aspects. Appl Ergon.

[b47-jhk-44-53] Knapik J, Ang P, Meiselman H, Johnson W, Kirk J, Bensel C, Hanlon W (1997). Soldier performance and strenuous road marching: influence of load mass and load distribution. Mil Med.

[b48-jhk-44-53] Knapik J, Jones S, Darakjy S, Hauret K, Bullock S, Sharp M, Jones B (2007). Injury rates and injury risk factors among U.S. Army wheel vehicle mechanics. Mil Med.

[b49-jhk-44-53] Knapik J, Reynolds K, Harman E (2004). Soldier load carriage: historical, physiological, biomechanical, and medical aspects. Mil Med.

[b50-jhk-44-53] Lambeek L, Van Tudler M, Swinkels I, Koppes L, Anema J, Van Mechelen W (2011). The trend in total cost of back pain in the Netherlands in the period 2002 to 2007. Spine.

[b51-jhk-44-53] Legg S (1985). Comparison of different methods of load carriage. Ergonomics.

[b52-jhk-44-53] Mäkelä J, Ramstad R, Mattila V, Pihlajamaki H (2006). Brachial plexus lesion after backpack carriage in young adults. Clin Orthop Relat Res.

[b53-jhk-44-53] Maki B (1997). Gait changes in older adults: predictors of falls or indicators of fear. J Am Geriatr Soc.

[b54-jhk-44-53] Marsh R, Ellerby D, Henry H, Rubenson J (2006). The energetic costs of trunk and distal-limb loading during walking and running in guinea fowl *Numida meleagris*. Organismal metabolism and biomechanics. J Exp Biol.

[b55-jhk-44-53] Matsuo T, Hashimoto M, Koyanagi M, Hashizume K (2008). Asymmetric load-carrying in young and elderly women: relationship with lower coordination. Gait Posture.

[b56-jhk-44-53] Mattila V, Parkkari J, Korpela H, Pihlajamaki H (2006). Hospitalization for injuries among Finnish conscripts in 1990–1999. Accid Anal Prev.

[b57-jhk-44-53] Mattila V, Sillanpaa P, Visuri T, Pihlajamaki H (2009). Incidence of low back pain hospitalisation during military service- an analysis of 387,070 Finnish young males. BMC Musculoskelet Disord.

[b58-jhk-44-53] Murdock G, Hubley-Kozey C (2012). Effect of a high intensity quadriceps fatigue protocol on knee joint mechanics and muscle activation during gait in young adults. Eur J Appl Physiol.

[b59-jhk-44-53] Murtezani A, Hundozi H, Orovcanec N, Berisha M, Meka V (2010). Low back pain predict sickness absence among power plant workers. IJOEM.

[b60-jhk-44-53] Myers S, Stergiou N, Pipinos I, Johanning J (2010). Gait variability patterns are altered in healthy young individuals during the acute reperfusion phase of ischemia-reperfusion. J Surg Res.

[b61-jhk-44-53] Niebuhr D, Scott C, Powers T, Li Y, Han W, Millikan A, Krauss M (2008). Assessment of recruitment motivation and strength study: preaccession physical fitness assessment predicts early attrition. Mil Med.

[b62-jhk-44-53] Niebuhr D, Scott C, Li Y, Bedno S, Han W, Powers T (2009). Preaccession fitness and body composition as predictors of attrition in U.S. Army recruits. Mil Med.

[b63-jhk-44-53] Oh J, Choi S (2007). Effects of the length of schoolbag string on gait posture. J Sport Leis Stud.

[b64-jhk-44-53] Pandolf K, Givoni B, Goldman R (1977). Predicting energy expenditure with loads while standing or walking very slowly. J Appl Physiol.

[b65-jhk-44-53] Parijat P, Lockhart T (2008). Effects of quadriceps fatigue on biomechanics of gait and slip propensity. Gait Posture.

[b66-jhk-44-53] Paschalis V, Theodorou A, Panayiotou G, Kyparos A, Patikas D, Grivas G, Nikolaidis M, Vrabas I (2013). Stair descending exercise using a novel automatic escalator: effects on muscle performance and health-related parameters. Plos ONE.

[b67-jhk-44-53] Pincus S (1991). Approximate entropy as a measure of system complexity. Proc Natl Acad Sci.

[b68-jhk-44-53] Punnett B, Greenridge D, Ramsey J (2007). Job attitudes and absenteeism: a study in the English speaking Caribbean. J World Bus.

[b69-jhk-44-53] Robertson D, Caldwell G, Hamill J, Kamen G, Whittlesey S (2004). Research Methods in Biomechanics.

[b70-jhk-44-53] Simpson K, Munro B, Steele J (2011). Backpack load affects lower limb muscle activity patterns of female hikers during prolonged load carriage. J Electromyogr Kinesiol.

[b71-jhk-44-53] Steenstra I, Anema J, Bongers P, de Vet H, Mechelen W (2009). Cost effectiveness of a multi-stage return to work program for workers on sick leave due to low back pain, design of a population based controlled trial. BMC Musculoskelet Disord.

[b72-jhk-44-53] Taanila H, Suni J, Pihlajamaki H, Mattila V, Ohrankammen O, Vuorinen P, Parkkari J (2010). Aetiology and risk factors of musculoskeletal disorders in physically active conscripts: a follow-up study in the Finnish Defence Forces. BMC Musculoskelet Disord.

[b73-jhk-44-53] Taanila H, Suni J, Pihlajamaki H, Mattila V, Ohrankammen O, Vuorinen P, Parkkari J (2009). Musculoskeletal disorders in physically active conscripts: a one-year follow-up study in the Finnish Defence Forces. BMC Musculoskelet Disord.

[b74-jhk-44-53] Waitzkin H, Noble M (2009). Caring for active duty military personnel in the civilian sector. Soc Med.

[b75-jhk-44-53] Whittfield J, Legg S, Hedderley D (2001). Schoolbag weight and musculoskeletal symptoms in New Zealand secondary schools. Ergonomics.

[b76-jhk-44-53] Woodhull A, Maltrund K, Mello B (1985). Alignment of the human body in standing. Eur J Appl Physiol.

